# Além dos Limites: O que 366 Maratonas Consecutivas Revelam sobre o Coração Humano

**DOI:** 10.36660/abc.20250231

**Published:** 2025-06-02

**Authors:** Fabricio Braga, Ricardo Stein

**Affiliations:** 1 Laboratório de Performance Humana Rio de Janeiro RJ Brasil Laboratório de Performance Humana, Rio de Janeiro, RJ – Brasil; 2 Universidade do Estado do Rio de Janeiro Departamento de Cardiologia Rio de Janeiro RJ Brasil Departamento de Cardiologia, Universidade do Estado do Rio de Janeiro (UERJ), Rio de Janeiro, RJ – Brasil; 3 Grupo de Pesquisa em Cardiologia do Exercício do Hospital de Clínicas de Porto Alegre (CardioEx-HCPA) Porto Alegre RS Brasil Grupo de Pesquisa em Cardiologia do Exercício do Hospital de Clínicas de Porto Alegre (CardioEx-HCPA), Porto Alegre, RS – Brasil; 4 Universidade Federal do Rio Grande do Sul Porto Alegre RS Brasil Universidade Federal do Rio Grande do Sul (UFRGS), Porto Alegre, RS – Brasil

**Keywords:** Corrida, Maratona, Coração do Atleta, Exercício Intenso, Limites

**"O corpo humano é capaz de adaptações incríveis — se lhe for dada a chance."**

— *Michael Joyner, fisiologista da Mayo Clinic e uma autoridade em desempenho de resistência*

A cultura ocidental sempre celebrou a busca por extremos físicos — desde feito épicos antigos até ultramaratonas modernas, atividades que envolvem a resistência sempre foi mais do que esporte: é narrativa, identidade e desafio aos limites percebidos. Completar uma única maratona já coloca um estresse substancial nos sistemas cardiovascular, metabólico e musculoesquelético. Correr uma maratona 366 vezes em 366 dias consecutivos muda o foco da fisiologia para a filosofia: Até onde o coração humano pode realmente ir?

O estudo de caso publicado nesta edição dos Arquivos Brasileiros de Cardiologia^1^ documenta um feito anteriormente inimaginável: um atleta amador brasileiro completou uma maratona completa todos os dias durante um ano — tudo sob supervisão médica sistemática. Mais que um Recorde Mundial do *Guinness* , isso é uma exploração em tempo real da adaptabilidade humana. O sistema cardiovascular do atleta permaneceu notavelmente estável ao longo do ano, com o consumo máximo de oxigênio (VO_2_ max), estrutura cardíaca e biomarcadores dentro de faixas fisiológicas, apesar do volume extremo de exercício.

Esse fenômeno se alinha com uma tendência mais ampla de recordes em provas de alta resistência que combinam desempenho com persistência. Desde os "50 *Ironmans* em 50 dias" até a corrida recorde de Márcio Villar de sete dias consecutivos na esteira^2^ — outro brasileiro listado no *Guinness Book of Records* — o mundo da resistência continua a reescrever seus próprios limites. Feitos como esse exigem foco inabalável, resiliência extrema e resistência mental quase sobre-humana. Eles também oferecem aos cardiologistas e cientistas do esporte oportunidades raras de observar a adaptação crônica de altíssima carga *in vivo.* ^3 , 4^

As adaptações fisiológicas à resistência prolongada incluem aumento do volume cardíaco, melhora da função endotelial e aumento do VO_2_ max. No entanto, estudos anteriores também sugeriram que o exercício de resistência excessivo pode estar associado à remodelação estrutural e maior incidência de arritmias ou calcificação das artérias coronárias.^5 , 6^ Neste caso, a ausência de remodelação adversa, apesar da carga sem precedentes, levanta questões críticas: a intensidade foi protetora? O suporte psicológico e nutricional foi essencial para prevenir a má adaptação?

O atleta treinou e competiu próximo ao seu primeiro limiar ventilatório, uma estratégia que pode ter mitigado o potencial de lesão miocárdica. Testes cardiopulmonares, ecocardiogramas e biomarcadores sanguíneos seriados revelaram um quadro de estabilidade. O coração do atleta, literalmente, resistiu.

No entanto, isso é mais que um triunfo fisiológico. É uma aula de preparação, apoio e variabilidade humana. Atletas de resistência de elite continuam a desafiar limites teóricos de desempenho — alguns modelos preveem um tempo de maratona de 1:57:58 como fisiologicamente possível.^4^ Da mesma forma, feitos como o descrito aqui nos forçam a reconsiderar onde o risco começa e termina. O limite entre adaptação e dano não é universal — é profundamente individual.

Existe também um toque poético nesta história. Muito similar a Sísifo, condenado a empurrar uma enorme pedra até o topo de uma montanha. Porém, toda vez que ele estava prestes a alcançar o cume, a pedra rolava de volta para baixo. No caso do maratonista em questão, este atleta enfrentou a mesma distância, dia após dia. Mas, ao contrário de Sísifo, ele escolheu este caminho — cada passo carregava sua própria lição. A monotonia tornou-se um método. A repetição, uma revelação. Em uma sociedade cada vez mais obcecada pelo imediatismo e atalhos, a consistência em longo prazo pode ser a forma final de rebelião.

Este editorial também levanta uma implicação científica essencial: estudos como este não devem ser vistos como glorificação do excesso, e sim como exploração do limite da saúde humana. Esforços de alta resistência exigem cuidados multidisciplinares — cardiologistas, fisiologistas, nutricionistas, psicólogos. Tais cuidados também deixam claras as limitações da estratificação de risco baseada em populações quando aplicada à fisiologia altamente individualizada.

Do ponto de vista clínico, este caso reforça o papel da triagem cardiovascular cuidadosa e do acompanhamento seriado em atletas que tentam desafios extremos. Lembra-nos que os limites fisiológicos não são limiares fixos, mas zonas flexíveis, moldadas pelo contexto, preparação e biologia.^7 , 8^

Finalmente, isso é um chamado para reformular a narrativa acerca do exercício. Embora a atividade física moderada continue sendo a base da saúde pública, histórias como essa nos desafiam a fazer melhores perguntas, desenhar estudos mais inteligentes e respeitar a variação individual. O excesso em um corpo pode ser adaptação em outro.

Os limites do corpo humano não são desenhados em livros didáticos. Eles são descobertos — às vezes uma maratona de cada vez.

Este estudo nos lembra: a resistência não é apenas sobre tempo ou distância. É sobre explorar onde o corpo se dobra, e onde — apenas talvez — ele não se quebra.
