# Supplementary material for: Além dos Limites: O que 366 Maratonas Consecutivas Revelam sobre o Coração Humano
Source: Arq Bras Cardiol. 2025 Jun 2;(5):e20250231. [Article in Portuguese] doi: 10.36660/abc.20250231 (PMC12212385; doi:10.36660/abc.20250231)
Supplement: Translation

# Translation: Beyond Limits: What 366 Consecutive Marathons Reveal about the Human Heart

**DOI:** 10.36660/abc.20250231i

**Authors:** Fabricio Braga, Ricardo Stein

**Affiliations:** 1 Rio de Janeiro RJ Brazil Laboratório de Performance Humana, Rio de Janeiro, RJ – Brazil; 2 Rio de Janeiro RJ Brazil Departamento de Cardiologia, Universidade do Estado do Rio de Janeiro (UERJ), Rio de Janeiro, RJ – Brazil; 3 Porto Alegre RS Brazil Grupo de Pesquisa em Cardiologia do Exercício do Hospital de Clínicas de Porto Alegre (CardioEx-HCPA), Porto Alegre, RS – Brazil; 4 Porto Alegre RS Brazil Universidade Federal do Rio Grande do Sul (UFRGS), Porto Alegre, RS – Brazil

**Keywords:** Running, Marathon, Athlete's Heart, Intense Exercise, Limits

**"The human body is capable of amazing adaptations — if given the chance."**

— *Michael Joyner, physiologist at the Mayo Clinic and a global authority on endurance performance*

Western culture has always celebrated the pursuit of physical extremes — from ancient epics to modern ultramarathons, endurance has always been more than sport: it is narrative, identity, and defiance of perceived limits. Completing a single marathon already places substantial stress on the cardiovascular, metabolic, and musculoskeletal systems. Doing a Marathon 366 times over 366 consecutive days shifts the focus from physiology to philosophy: Just how far can the human heart truly go?

The case study published in this issue of *Arquivos Brasileiros de Cardiologia* ^1^documents a feat previously unimaginable: a Brazilian amateur athlete completed a full marathon every single day for a year — all under systematic medical supervision. More than a Guinness World Record, this is a real-time exploration of human adaptability. The athlete's cardiovascular system remained remarkably stable throughout the year, with maximal oxygen uptake (VO_2_max), cardiac structure, and biomarkers within physiological ranges, despite the extreme exercise volume.

This phenomenon aligns with a broader trend of ultra-endurance records that blend performance with persistence. From the "50 Ironman's in 50 days" to Márcio Villar's record-breaking run of seven consecutive days on a treadmill^2^— another Brazilian listed in the *Guinness Book of Records* — the endurance world continues to rewrite its own limits. Feats like this demand unbreakable focus, fierce resilience, and near-superhuman mental endurance. They also offer cardiologists and sports scientists rare opportunities to observe chronic, high-load adaptation *in vivo* .^3 , 4^

Physiological adaptations to prolonged endurance include increased cardiac volume, improved endothelial function, and enhanced VO_2_max. However, previous studies have also suggested that excessive endurance exercise may be associated with structural remodeling and higher incidence of arrhythmias or coronary artery calcification.^5 , 6^In this case, the absence of adverse remodeling despite the unprecedented load raises critical questions: was the intensity protective? Was psychological and nutritional support essential in preventing maladaptation?

The athlete trained and competed close to his first ventilatory threshold, a strategy that may have mitigated the potential for myocardial injury. Serial cardiopulmonary tests, echocardiograms, and blood biomarkers revealed a picture of stability. The athlete's heart, quite literally, endured.

Yet this is more than a physiological triumph. It is a lesson in preparation, support, and human variability. Elite endurance athletes continue to challenge theoretical performance limits — some models predict a marathon time of 1:57:58 as physiologically possible.^4^Similarly, feats like the one described here force us to reconsider where risk begins and ends. The boundary between adaptation and damage is not universal — it is deeply individual.

There's also cultural poetry in this story. Much like Sisyphus, condemned to push a huge rock up to the top of a mountain. Yet, every time he was about to reach the summit, the rock would roll back down. In the case of the marathon runner in question, this athlete faced the same distance, day after day. But unlike Sisyphus, he chose this path — every step carried its own lesson. Monotony became a method. The repetition, a revelation. In a society increasingly obsessed with immediacy and shortcuts, long-term consistency may be the ultimate form of rebellion.

This editorial also raises an essential scientific implication: studies like this should not be seen as glorifying excess, but rather as exploring the outer envelope of human health. Ultra-endurance efforts demand multidisciplinary care — cardiologists, physiologists, nutritionists, psychologists. They also highlight the limitations of population-based risk stratification when applied to highly individualized physiology.

From a clinical perspective, this case reinforces the role of careful cardiovascular screening and serial follow-up in athletes attempting extreme challenges. It reminds us that physiological limits are not fixed thresholds but flexible zones, shaped by context, preparation, and biology.^7 , 8^

Finally, this is a call to reframe the narrative around exercise. While moderate physical activity remains the foundation of public health, stories like this challenge us to ask better questions, design smarter studies, and respect individual variation. Excess in one body may be adaptation to another.

The limits of the human body are not drawn in textbooks. They are discovered — sometimes one marathon at a time.

This study reminds us: endurance is not just about time or distance. It is about exploring where the body bends, and where — just maybe — it does not break.
